# A flexible multiplexed immunosensor for point-of-care in situ wound monitoring

**DOI:** 10.1126/sciadv.abg9614

**Published:** 2021-05-21

**Authors:** Yuji Gao, Dat T. Nguyen, Trifanny Yeo, Su Bin Lim, Wei Xian Tan, Leigh Edward Madden, Lin Jin, Ji Yong Kenan Long, Fazila Abu Bakar Aloweni, Yi Jia Angela Liew, Mandy Li Ling Tan, Shin Yuh Ang, Sivagame D/O Maniya, Ibrahim Abdelwahab, Kian Ping Loh, Chia-Hung Chen, David Laurence Becker, David Leavesley, John S. Ho, Chwee Teck Lim

**Affiliations:** 1 Institute for Health Innovation & Technology (iHealthtech), National University of Singapore, Singapore 117599, Singapore.; 2 Integrative Sciences and Engineering Programme, NUS Graduate School, National University of Singapore, Singapore 119077, Singapore.; 3 Department of Electrical and Computer Engineering, National University of Singapore, Singapore 117583, Singapore.; 4 Department of Biomedical Engineering, National University of Singapore, Singapore 117583, Singapore.; 5 Nursing, Singapore General Hospital, Singapore 168753, Singapore.; 6 Lee Kong Chian School of Medicine, Nanyang Technological University, Singapore 308232, Singapore.; 7 Department of Chemistry, National University of Singapore, Singapore 117543, Singapore.; 8 Department of Biomedical Engineering, City University of Hong Kong, 83 Tat Chee Avenue, Kowloon, Hong Kong, China.; 9 Skin Research Institute of Singapore, Agency for Science, Technology and Research (A*STAR), Singapore 138648, Singapore.; 10 Mechanobiology Institute, National University of Singapore, Singapore 117411, Singapore.

## Abstract

Chronic wounds arise from interruption of normal healing due to many potential pathophysiological factors. Monitoring these multivariate factors can provide personalized diagnostic information for wound management, but current sensing technologies use complex laboratory tests or track a limited number of wound parameters. We report a flexible biosensing platform for multiplexed profiling of the wound microenvironment, inflammation, and infection state at the point of care. This platform integrates a sensor array for measuring inflammatory mediators [tumor necrosis factor–α, interleukin-6 (IL-6), IL-8, and transforming growth factor–β1], microbial burden (*Staphylococcus aureus*), and physicochemical parameters (temperature and pH) with a microfluidic wound exudate collector and flexible electronics for wireless, smartphone-based data readout. We demonstrate in situ multiplexed monitoring in a mouse wound model and also profile wound exudates from patients with venous leg ulcers. This technology may facilitate more timely and personalized wound management to improve chronic wound healing outcomes.

## Teaser

An integrated smart microfluidic dressing with a multibiomarker immunosensor can provide in situ wound status profiling on site.

## INTRODUCTION

Chronic wounds are debilitating disorders that can cause severe distress to afflicted patients. Globally, they pose an increasing social and financial burden to health care systems due to the increasing aging population (*1*, *2*). For example, venous ulcers require long-term therapeutics to heal, with a prevalence of up to 15% of people aged over 70 and recurrence rates varying from 54 to 78% (*3*, *4*). Chronic wounds result from failure to undergo the natural healing process due to multiple environmental and physiological factors. These factors are reflected in the composition of the wound exudate fluid (*5*), which exhibits a dynamic mixture of cytokines, growth factors, and microorganisms during the progression of wound healing.

Clinical assessment of wounds currently relies on planimetry to qualitatively score features such as slough reduction, granulation tissue formation, and reepithelialization (*6*). At present, quantitative profiling of the biochemical parameters is generally limited to downstream laboratory testing, such as enzyme-linked immunosorbent assays (ELISAs) (*2*). A noninvasive, point-of-care wound care device that is capable of in situ surveillance of the wound biomarkers can provide timely analysis for more effective diagnosis and treatment (*7*–*9*). Current flexible sensors designed for wound care are capable of monitoring a limited set of parameters, such as pH (*10*, *11*), temperature (*11*), oxygen (*12*), uric acid (*13*), and impedance (*14*). Beyond these limited markers, indicators of inflammatory mediators and bioburden are also of substantial clinical value. For example, cytokines and growth factors are well-established indicators of inflammation during ulcer formation (*1*). The microbial composition of the wound is also an essential feature of chronic wounds, having been implicated in the inhibition of healing through sustained inflammation, proteolysis, and endothelial dysfunction (*1*, *2*). On the basis of these indicators, panels of biomarkers have been proposed for classifying the healing status of wounds (*15*). These panels that provide information about the wound healing status can better guide clinical wound management as compared to just through visual inspection or single-marker measurement.

**Fig. 1:**
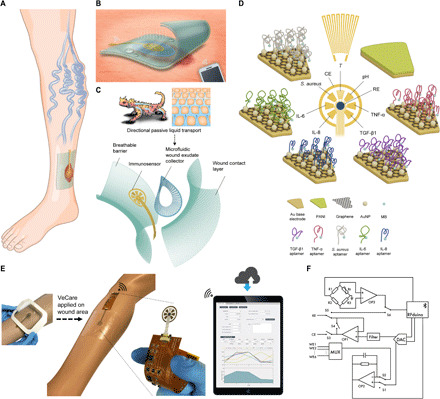
Schematic of a multiplexed immunosensing system for chronic wound monitoring. (**A**) Illustration of a biomarker analytical dressing applied onto an open wound of patients with venous ulcer for in situ wound surveillance. (**B**) Illustration of a thin, soft, biomarker analytical dressing that allows normal skin function by letting oxygen in and moisture vapor out. Measurement data were wirelessly transmitted to a paired mobile system over Bluetooth Low Energy. (**C**) Envisioned biomarker analytical dressing constituting a perforated wound contact layer, a microfluidic wound exudate collector, an immunosensor, and a breathable barrier. The microfluidic collector was inspired by the skin of Texas horned lizard enabling predetermined flow direction toward the lizard’s snout defying gravity. (**D**) Schematic of the immunosensor for detection of TNF-α, IL-6, IL-8, TGF-β1, *S. aureus*, pH, and temperature. PANI, polyaniline; MB, methylene blue; RE, reference electrode; CE, counter electrode. (**E**) VeCare prototype for envisioned chronic wound monitoring. The prototype was applied to a leg dummy as a demonstration. The immunosensor interfacing with a wireless portable analyzer fabricated on a FPCB. A mobile application providing a GUI as a one-stop patient’s profiles, medical records, data recording, data analysis, and result visualization system is shown. (**F**) Hardware block diagram for the VeCare platform. WE1, working electrode 1; MUX, multiplexer.

Here, we report a flexible microfluidic multiplexed immunosensing platform for the point-of-care quantitative assessment of a panel of biomarkers that can enable in situ profiling of the wound microenvironment, inflammation, and infection state. This platform incorporates a number of advances over prior, single-marker sensors: (i) microdrop functionalization technique; (ii) microfluidic layouts and materials for sensing and fluid collection; (iii) previously unidentified aptamer sequences; and (iv) integration with wireless, flexible electronics. Using this platform, we demonstrate a venous ulcer care device, termed VeCare, that interfaces directly with wounds in the form of a bioanalytical dressing and quantifies a broad panel of healing biomarkers, including inflammatory mediators, bacterial load, and physicochemical parameters (Fig. 1D). The biomarkers selected for the panel include tumor necrosis factor–α (TNF-α), interleukin-6 (IL-6), and IL-8, which are elevated in wound fluids obtained from nonhealing ulcers as compared to healing ulcers (*16*–*18*). To assess the status of dermal healing in chronic skin lesions, the panel also includes transforming growth factor–β1 (TGF-β1), which plays a key role in regulating dermal fibroblast phenotype and function and has been clinically observed in elevated concentrations in exudates from venous ulcers (*19*). The physicochemical markers in our panel include pH, temperature, and bacterial load (*20*, *21*). The pH of wound exudate is an important biochemical indicator of wound healing status: Hard-to-heal wounds generally exhibit alkaline pH, ranging from 7.15 to 8.93 (*22*–*24*). Wound temperature provides information on inflammation and infection: Wounds with elevated temperature tend to heal more slowly (*7*). *Staphylococcus aureus*, a predominant species in all types of chronic wound samples, can be a useful biomarker for bioburden on wounds (*25*).

To facilitate real-time clinical feedback, we also designed a portable wireless analyzer to interface with the immunosensor, along with an accompanying application containing a graphical user interface (GUI) to assist the management of patient’s profile and medical records, while facilitating data collection, analysis, and visualization. The proposed VeCare is designed to be easily used by clinicians for in situ wound fluid analysis and prognosis, permitting clinical management decisions to be provided with time sensitivity, reducing diagnostic delays and clinical consults, and delivering substantial cost savings for all stakeholders. To demonstrate the capability of in situ wound monitoring, we placed the VeCare platform in direct contact with a full-thickness excisional wound in a mouse model, followed by in situ multibiomarker assessment over the wound healing process. We further demonstrated the biocompatibility of the platform by performing behavioral and histological assessments. As an example of potential clinical application, the VeCare device was applied to assay TNF-α, IL-6, IL-8, TGF-β1, *S. aureus*, and pH in wound fluids collected from five patients with active venous ulcers, once a week for five consecutive weeks.

## RESULTS

### Design of the multiplexed immunosensing platform

Figure 1A shows a biomarker analytical dressing containing an immunosensor applied onto an open wound of patients with venous ulcer for in situ wound surveillance. The dressing is composed of a perforated wound contact layer, a microfluidic wound exudate collector, an immunosensor, and a breathable barrier (Fig. 1C). The function of the perforated wound contact layer is to protect the immunosensor from direct contact with the wound bed, minimizing disruption to the granulating tissue. The barrier allows normal skin function by letting oxygen in and moisture vapor out (Fig. 1B). The transparency of the dressing substrates allows convenient observation and evaluation of the wound in situ during application: surface area and color of the exudate. Figure 1E presents a VeCare prototype for venous ulcer monitoring. A portable wireless analyzer was fabricated and assembled on a flexible printed circuit board (FPCB) to interface with the immunosensor (Materials and Methods). Figure 1F illustrates the circuit block diagram describing the hardware design. The portable electrochemical analyzer is designed to manage signal transduction and perform electrochemical measurements. Measurement data are wirelessly transmitted to a paired mobile device using Bluetooth Low Energy (BLE). We have developed an accompanying application, containing a GUI to assist the management of patient’s profiles and medical records, while facilitating data collection, analysis, and visualization (Materials and Methods; fig. S13 and movies S9 to 11).

### Design of the microfluidic wound exudate collector

**Fig. 2:**
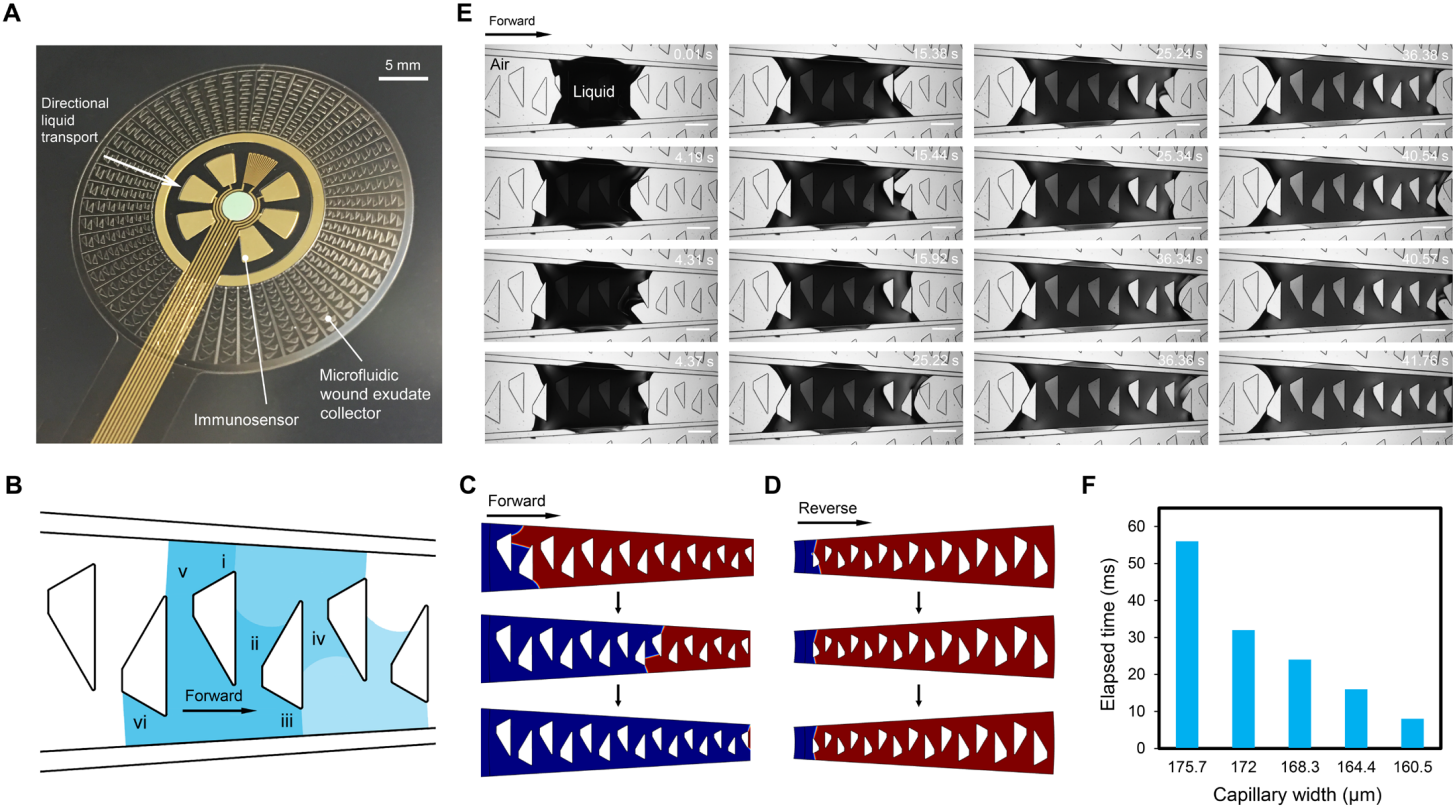
Microfluidic wound exudate collector enabling directional liquid transport. (**A**) A biomimetic passive microfluidic collector formed by a polar array of interconnected half-open, sawtooth-shaped capillary channels with a decreasing width from 200 to 160 μm was fabricated on top of the base electrodes. (**B**) Mechanism of a directional liquid transport system exploiting the interconnection of adjacent sawtooth-shaped capillary channels. (**C** and **D**) COMSOL simulation of liquid transport in the interconnected capillary channels with decreasing width in forward and reverse directions with time, respectively (blue, liquid; red, air). (**E**) Dynamics of liquid transport process of a biomimetic prototype at different time points (scale bars, 500 μm). (**F**) Elapsed time of the liquid transport in capillary channels with decreasing width.

Efficient wound fluid capture and delivery is essential for accurate in situ biomarker detection. To ensure efficient wound fluid collection, we incorporated a microfluidic layer capable of guiding the wound fluids to the sensing area. We surveyed a variety of natural structures where passive directional liquid transport system is formed via wettability gradient (e.g., back of desert beetles), curvature gradient (e.g., shorebird beaks, spider silk, and cactus spines), and asymmetric structures (e.g., skin of desert lizards and peristome of pitcher plants) (*26*). Notably, we were inspired by the skin of Texas horned lizard (*Phrynosoma cornutum*) that enables predetermined directional fluid flow toward the lizard’s snout defying gravity (Fig. 1C), for our reference design of a biomimetic passive microfluidic wound exudate collector. The directional liquid transportability of *P. cornutum*’s skin is ascribed to a network of microstructures that forms a special capillary system between the scales (*27*). On the basis of a theoretical model derived from this capillary network, the passive microfluidic wound exudate collector was designed with an annular pattern (Fig. 2A). The pattern is composed of a polar array of interconnected half-open, sawtooth-shaped capillary channels with a decreasing width from 200 to 160 μm (outer to inner). The interconnection of adjacent sawtooth-shaped capillary channels facilitates continuous flow in the forward direction (i.e., toward the sensing area) but inhibits liquid transport in the reverse direction. Specifically, in the forward direction, the liquid stops at capillary i because of abrupt widening with infinite meniscus radius, while continuously flowing to capillary ii, where it merges with the stopped fluid at capillary i and flows forward to capillary iv. Similarly, the merged liquid will coalesce with the stopped liquid at capillary iii and keep on flowing to subsequent capillaries. On the other hand, the liquid flow stops at widening sections v and vi in the reverse direction (Fig. 2B). Therefore, a directional liquid transport system is formed.

To demonstrate this working principle, we built the directional liquid transport system models (Materials and Methods). Simulation of the liquid transport in the interconnected capillary channels with decreasing width was conducted in both forward and reverse directions, as shown in Fig. 2 (C and D). The liquid passed through in the forward direction; it stopped completely at the starting point in the reverse direction. In addition, the liquid transport performance with respect to capillary channel of widths 160 and 200 μm was also simulated. It was observed that at width of 200 μm, the liquid failed to flow in the forward direction (fig. S1A), while a continuous flow was observed under the width of 160 μm (fig. S1C). On the other hand, the liquid failed to overcome the starting point in the reverse direction in both capillary channel widths (fig. S1, B and D). The dynamic processes are shown in movies S1 to S6. On the basis of these simulation results, the capillary channel with decreasing width can ensure efficient and continuous directional liquid transport toward the sensing area.

Experimental results obtained using a biomimetic prototype verified the simulation results. Specifically, a droplet (2 μl) of soapy water, which has a similar contact angle as human serum, was applied to the middle part of the directional liquid transport system. Figure 2E illustrates the dynamic passive liquid transport process (Materials and Methods; movie S7) at different times. By consecutively using the interconnected capillary channels, the liquid was transported to the sensing area, whereas the movement in the reverse direction was inhibited. We also observed that decreasing capillary channel width required shorter liquid transport time in the capillary channels (Fig. 2F). The average flow rate in the forward direction was ~0.43 mm^3^ s^−1^. The design of the directional liquid transport system provided additional ~180% wound fluid capture and delivery to the sensor within 130 s, ensuring reliable sensing performance regardless of the ulcer shape or size.

### Design and characterization of the immunosensor

The immunosensor was designed to measure multiple biophysicochemical parameters of sampled wound fluid based on an electrochemical system. It contained a polar array of petal-shaped working electrodes sharing one Ag/AgCl reference electrode at the center and one Au counter electrode at the periphery (Materials and Methods; Fig. 1D), forming a compact circular layout ideal for microvolume analysis. The sensing elements of the TNF-α (*28*), IL-6 (*29*), IL-8 (*30*), and TGF-β1 (*31*) electrodes were based on aptamer-analyte affinity, while the binding affinity of the *S. aureus* electrode was between the aptamer and specific epitopes at the surface of bacteria cell wall (*32*).

Prior flexible aptamer-based biosensor functionalization methods relied on fabrication techniques relating to a single biomarker (table S1) (*33*–*37*). To overcome the challenge of integrating multiple aptasensing modalities within a compact sensing area, we used a microdrop procedure with the aid of microwells to functionalize each working electrode with different sensing elements. An optimized height of the microwell (20 μm) enabled independent drop casting, aptamer immobilization, and passivation, while also protecting the immobilized aptamers and captured targets from being scratched off (Materials and Methods).

To optimize the performance of microelectrodes, we modified each aptamer-based electrode with a thin layer of electrochemically exfoliated graphene–gold nanoparticles (AuNPs-GP) nanocomposite (Materials and Methods). Electrochemically exfoliated graphene, referred to as “graphene,” has excellent properties such as high crystallinity, high conductivity, and degree of low oxidation. In addition, gold nanoparticles provide high current density, enhanced electron mobility, and fast mass transport (*38*). The morphology of the AuNPs-GP was characterized from field-emission scanning electron microscope (FESEM) images (fig. S2B). The Raman spectra of graphene and AuNPs-GP nanocomposite are shown in fig. S2C (Materials and Methods). The D-to-G band peak intensity ratio (*I*_D_/*I*_G_) reflects the degree of disorder in the graphitic material (*39*). The *I*_D_/*I*_G_ value of AuNPs-GP (0.93) was slightly larger than that of graphene (0.91), indicating additional defects introduced by AuNPs to the nanocomposite.

Qualitative analysis of the AuNPs-GP modified electrodes was conducted using cyclic voltammetry (CV) at different scan rates, as illustrated in fig. S3A. The anodic-to-cathodic peak current ratios (*I*_pa_/*I*_pc_) was in the range of 0.92 to 1.15, indicating the quasi-reversibility of the system (*39*, *40*). Theoretically, the peak current is proportional to the square root of the scan rate (*39*). Figure S3B shows the variation of anodic and cathodic peak current with respect to the square root of the scan rate, with regression coefficients of 0.9867 and 0.9903, respectively. Good linearity indicated diffusion-controlled processes at the electrode.

**Fig. 3:**
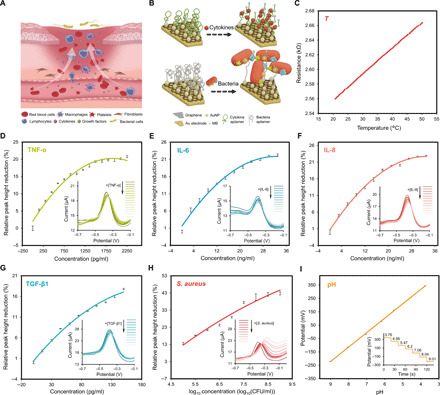
Characterization of the TNF-α, IL-6, IL-8, TGF-β1, *S. aureus*, and pH sensors. (**A**) Illustration of a microenvironment of venous ulcers. (**B**) Schematic of the sensing mechanism of the aptasensors for cytokine and bacteria detection, respectively. (**C**) Calibration of the resistance of the temperature sensor versus the temperature. (**D** to **H**) Variations in relative peak height reduction of the TNF-α, IL-6, IL-8, TGF-β1, and *S. aureus* sensors versus the concentration of corresponding targets in serum, respectively. Error bars denote the SD of the mean derived from three scans under same conditions. The insets of (D to H) show SWV scans of the TNF-α, IL-6, IL-8, TGF-β1, and *S. aureus* sensors when challenged with different analyte concentrations, respectively. (**I**) Calibration of the OCP of the pH sensor versus pH values in serum. Error bars denote the SD of the mean over a 20-s span under same conditions. The inset of (I) shows the real-time OCP of the pH sensor for different pH values.

We designed novel aptamer sequences (Materials and Methods) modified with methylene blue (MB), a redox probe, at one end and a thiol group at the other end for covalent binding to AuNPs. As illustrated in Fig. 3B, in the absence of an analyte, MB is in proximity to the AuNPs-GP–modified electrodes, allowing electron transfer. A Faradaic current can be detected electrochemically. Upon target binding, the hairpin structure of the aptamer undergoes a conformational change during which MB moves away from the electrodes, causing a decreased redox current. Owing to the sensing mechanism (*28*, *41*), the redox probe–modified aptasensors require no additional reagent to accomplish the electrochemical measurement in a single step, which makes it appropriate for in situ determination and independent from downstream analysis. Having advantages of good chemical and thermal stability, high affinity, good selectivity, and nonimmunogenicity (*40*–*43*), the aptamer serves as a vital role in establishing a biocompatible molecular analytical dressing. Following aptamer immobilization, we passivated the electrode surface with 6-mercapto-1-hexanol (MCH) to inhibit nonspecific adsorption.

The stepwise assembly of different layers on the working electrode was validated by electrochemical impedance spectroscopy (EIS) (Materials and Methods; fig. S3C). The AuNPs-GP–modified electrode had a lower charge transfer resistance (*R*_ct_) (3.17 kilohm) than the bare gold electrode (5.29 kilohm), indicating enhanced electron transfer kinetics at the electrode interface with higher electroactive surface area (*39*). The immobilization of aptamer and MCH led to an increase of *R*_ct_ to 14.4 and 17 kilohm, respectively. The electron mobility was hindered by the immobilized substance (*44*). The presence of an analyte further blocked the electron transfer indicated by a raised *R*_ct_ of 18 kilohm. Figure S3D reports the CV analysis of different steps where redox peak current is the indicator of conductivity (*39*). The results were consistent with those from EIS, verifying that the electrode preparation was successful and functional.

The aptamer-based sensors were characterized using square wave voltammetry (SWV) to monitor variations of peak current height associated with the MB redox tag distance to the electrode. Owing to its similar molecular composition to wound fluid, we used serum to mimic wound exudates. The aptamer density of 10 μM was applied to ensure distinguishable peak heights (fig. S4A) and to optimize the signal-to-noise ratio (SNR) (fig. S4B), where signal denotes the peak height and noise denotes the SD of the signal. Studies on the incubation process revealed that approximately 30 min is required for the aptamer-target binding to establish equilibrium (fig. S4, C to G). The performance of each aptasensor against analyte with different concentrations is shown in the inset of Fig. 3 (D to H, respectively) (Materials and Methods). The peak current height of each aptasensor was observed to decrease with increased target concentration. The relative reduction of peak height normalized to peak height against no analyte with analyte concentration is exhibited in Fig. 3 (D to H, respectively). Quadratic regression lines exhibit good monotonicity of the TNF-α (*R*^2^ = 0.9798), TGF-β1 (*R*^2^ = 0.9931), IL-8 (*R*^2^ = 0.9958), IL-6 (*R*^2^ = 0.9882), and *S. aureus* (*R*^2^ = 0.9712) sensors. Notably, the concentration ranges of TNF-α (0 to 2 ng ml^−1^), TGF-β1 (0 to 150 pg ml^−1^), IL-8 (0 to 30 ng ml^−1^), and IL-6 (0 to 30 ng ml^−1^) were based on levels reported in wound fluids from patients with venous ulcer (*16*, *17*, *19*, *45*), combined with ELISA results from clinical samples used for this study. Similarly, the range of *S. aureus* [0 to 1 ×10^9^ colony-forming units (CFU) ml^−1^] was selected on the basis of microbial loads reported in wounds (*46*, *47*), together with CFU enumerations from this study. Our cytokine sensors were demonstrated to have good selectivity, minimal interference (fig. S5, A to D), and good reproducibility (fig. S4H).

The working electrode for pH sensing is based on polyaniline (PANI) polymer (Materials and Methods) (*10*, *48*). The open-circuit potential (OCP) change was used to monitor pH variations. As shown in the inset of Fig. 3I, the potential of the pH sensor remains stable until the pH level changes. A decrease of potential was found with fluid samples changing from acidic to alkaline. The characterization of the pH sensor in the serum is illustrated in Fig. 3I. The sensitivity, defined as the relative change in OCP per unit of pH values normalized to the OCP at pH 3.76, is −31.402% [pH]^−1^. Having demonstrated good linearity (*R*^2^ = 0.9997, derived from the linear regression line), the pH sensor is also characterized by good repeatability (fig. S6A) and reproducibility (fig. S6B). The performance of the pH sensor was also validated with plasma yielding similar results (fig. S6, C to F). The embedded temperature sensor is based on a thermally responsive resistor (Materials and Methods) (*49*). The aptasensors and pH sensor showed good long-term stability over 4 weeks with drifts less than 5% (Materials and Methods; fig. S7), offering the advantage over antibody-based biosensors, which are easily inactivated because of antibody denaturation (*35*, *41*, *42*).

The characterization of the temperature sensor is presented in Fig. 3C. The sensitivity, defined as the relative change in resistance per unit of temperature normalized to the resistance at 20°C, is 0.1384% °C^−1^ with a good linearity of *R*^2^ = 0.9998. Note that the working ranges of the pH sensor (pH 4 to 9) and the temperature sensor (20° to 50°C) were considered to ensure coverage of the pH and temperature variations in a wound fluid environment (*10*).

The immunosensor embedded in the biomarker analytical dressing is designed with a sensing area (diameter of 16 mm) appropriate for use with most venous ulcers. Nevertheless, the size of the immunosensor can be adjusted proportionally (fig. S8) to fulfill a variety of other potential applications (e.g., acute trauma, surgical wounds, psoriasis, and eczema). The limit of resizing was explored to be 8-mm diameter of the sensing area due to the limitation of volume that a microwell can hold for independent aptamer immobilization in the multiplexed aptasensing system. Figure S8A shows the minimum volume required for the immunosensor under different sizes. The SNR loss when the sensor was resized to a smaller one (diameter of 8 mm) was observed to be less than 5% (fig. S8C).

### In situ wound monitoring and biocompatibility study in mice models

**Fig. 4:**
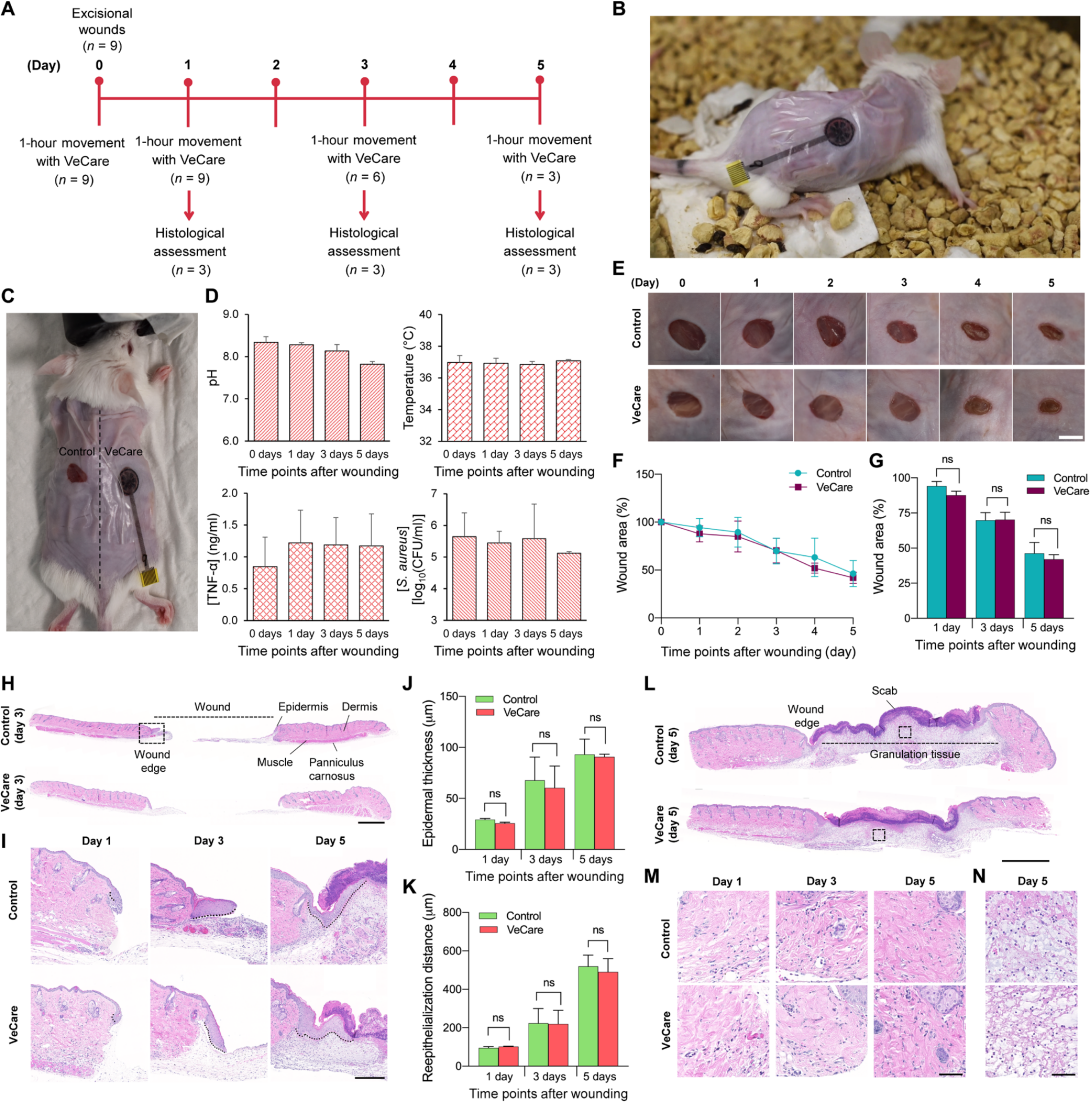
In situ monitoring on wound healing and biocompatibility in mouse wound model. (**A**) Wound monitoring study design. (**B**) Photograph of a freely moving mouse with an immunosensor mounted on the skin wound. (**C**) Photograph of the excisional wounds. The immunosensor is in direct contact with right wound, while the left wound acts as a control. (**D**) In situ assessment of pH, temperature, mouse TNF-α, and *S. aureus* by the immunosensor. (**E** and **F**) Images of the wounds (scale bar, 5 mm) and changes in wound area from days 0 to 5. (**G**) Comparison of wound area on days 1, 3, and 5 (cumulative total of 2, 3, and 4 hours of sensor contact). (**H** and **I**) H&E images (20× stitches) of whole full-thickness wounds on day 3 and wound edges on days 1, 3, and 5, respectively (scale bars, 1000 and 250 μm). Black dotted lines show reepithelialization. (**J** and **K**) Comparison of epidermal thickness and reepithelialization distance, respectively. (**L** to **N**) H&E images of whole wounds on day 5, dermis at wound edges on days 1, 3, and 5, and area of granulation tissue on day 5, respectively (scale bars, 1000, 250, and 250 μm). Images are typical representations across all mice. Statistical comparisons use Wilcoxon signed-rank test (ns, nonsignificant result). Error bars show SD (D and F) or SE (G, J, and K) of the mean.

To demonstrate the utility of the platform for in situ wound monitoring, we performed longitudinal wound monitoring in mice models (Materials and Methods). Briefly, two bilateral full-thickness excisional wounds were made on day 0, equidistant from the midline and spaced on either side of the dorsum (Fig. 4A). An immunosensor was in direct contact with the right wound (Fig. 4C), while the left wound was used as a control. Subjects were allowed to freely move for 1 hour with the attached immunosensor, followed by in situ wound monitoring. The presence of the immunosensor appeared to be well tolerated, with no observed signs of discomfort during freely moving behavior or any excessive scratching of the sensor-covered wound (Fig. 4B and movie S8). In all cases, the immunosensor remained functional after 1-hour contact with the wound. Figure 4D illustrates the longitudinal assessment of pH, temperature, mouse TNF-α, and *S. aureus* at time of wounding [day 0 (*n* = 9)] as well as at day 1 (*n* = 9), day 3 (*n* = 6), and day 5 (*n* = 3) after wounding. Longitudinal pH measurements show that the pH at the wound site decreases by 6% on day 5 compared to day 0. This decrease corresponded with reepithelialization of the wound, which is associated with hypoxia and lactic acid production (*24*). The immunosensor measurement also revealed a significant increase (give 44% increase) in TNF-α from days 0 to 1 corresponding to the inflammatory response after wounding (*50*). In contrast, the temperature and level of *S. aureus* measured at the wound were constant throughout the healing duration, which is consistent with the absence of infection as assessed by visual inspection (*7*, *51*). These results demonstrated that the immunosensor allows for in situ multibiomarker profiling of wound fluid over the duration relevant to wound healing.

Histological examination of the wound site further demonstrates the biocompatibility of the immunosensor. No apparent signs of adverse reactions (e.g., redness, swelling, and degeneration) were observed on the skin surface that was in contact with the immunosensor over 5 days (Fig. 4E). Figure 4 (F and G) shows no cumulative effect of the sensor placement on the rate of wound closure. The reepithelialization distance and nascent epidermal thickness were measured from hematoxylin and eosin (H&E) tissue section images (Fig. 4, H and I) to assess the effect of sensor placement on wound healing (*52*). No significant difference was observed between control wounds and wounds that had sensor contact (Fig. 4, J and K). Qualitative assessment of the immune cells in the dermis at the wound edge, identified by cell morphology and polymorphonuclear presence, suggested no difference in infiltration at all time points of sensor contact (Fig. 4, L and M) (*53*). Figure 4N reveals no difference in granulation tissue maturation or levels of cellularity (*54*). The behavioral, visual, and histological assessment in the wound healing mice models demonstrated the biocompatibility of the immunosensor for in situ wound surveillance.

### Clinical study on wound exudates from patients with venous ulcer

**Fig. 5:**
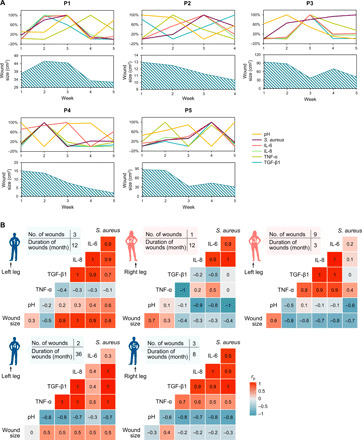
Sensor-derived data analysis of wound exudate samples from patients with venous ulcer. (**A**) Weekly assessment of pH, *S. aureus*, IL-6, IL-8, TNF-α, and TGF-β1 by the immunosensor for each patient. The axes represent independent scales for each of the quantified parameter, varying from 0% for the lowest level to 100% for the highest level. Weekly changes of the wound size are shown along with the biomarker assessment. (**B**) Patient-specific correlation matrices of parameters assessed by the immunosensor (pH, *S. aureus*, IL-6, IL-8, TNF-α, and TGF-β1) and the wound size over a 5-week period. The total number and duration of wounds (in months) are shown in the table for each patient. The color of standing person illustration indicates the gender of the patient (blue, male; pink, female). Scale bar represents Pearson’s correlation coefficient (*r*_p_).

To assess the clinical application of the VeCare immunosensor, we used this platform to analyze wound exudate from venous ulcers to objectively observe wound bed characteristics and bioburden (Materials and Methods). Briefly, wound exudates from five patients (P1 to P5) with clinically diagnosed nonhealing venous ulcers were consecutively collected once a week for 5 weeks. The wound exudates were assessed using the VeCare platform. Figure 5A illustrates the longitudinal changes of TNF-α, IL-6, IL-8, TGF-β1, *S. aureus*, pH, and wound sizes. It is clear that the readings for each biomarker varied over the period of study. While each patient’s wound fluids exhibited individually unique longitudinal profiles, some common features were evident. For example, wound fluids in P2 and P3 became less alkaline during weeks 1 to 3 and weeks 2 to 5, respectively, suggesting that their wounds were positively responding to the therapies for those periods (*22*, *23*). P2 (week 3), P4 (week 2), and P5 (week 4) experienced elevated load of *S. aureus* and exhibited more alkaline wound fluids in their subsequent week of visit. These observations were consistent with the reported associations between wound infection and pH (*20*, *21*). P2 who exhibited high levels of *S. aureus* had corresponding elevated levels of IL-6 and IL-8 at week 3. Similar responses were observed in P1 (week 3), P3 (week 3), P4 (week 2), and P5 (week 4) with elevated *S. aureus*, IL-6, IL-8, and TNF-α levels, consistent with the observations in keratinocytes (*55*). P1 (week 2) and P5 (week 4) who showed an increase of wound size were observed to have elevated levels of IL-6 and IL-8, consistent with the observations in a pilot study of 10 refractory venous ulcers (*56*). Apart from this, the inflammation and colonization tendencies in P1 (week 3), P2 (week 3), P4 (week 2), and P5 (week 4) were reduced in the following week, indicating that the clinical interventions (e.g., topical dressings impregnated with antiseptic) seem to be effective at reducing microorganism loads. These multibiomarker profiles can provide comprehensive wound-specific parameters and inform clinical decisions on modality and duration of treatment.

To assess the effect of wound exudates on the sensor performance, we used analytes with different high concentrations or pH values to challenge the immunosensor after use. Figure S9 (A to E) shows the performance of each aptasensors, respectively. The peak current height of each aptasensor was observed to further decrease with increased target concentration, as shown in the insets of fig. S9 (A to E, respectively). Figure S9F exhibits consistent performance of the pH sensor with a newly prepared one. The performance of the immunosensor was shown not to be compromised after exposure to the wound exudates.

The biomarkers, as earlier measured by the immunosensor, were further independently assessed using conventional approaches. The levels of cytokines, pH, and *S. aureus* were determined using multiplex ELISA, pH meter, and coagulase-positive *Staphylococci* enumeration, respectively (Materials and Methods). Figure S10 presents longitudinal changes in the level of each biomarker for each patient. These measurements exhibited similar features as the sensor readings, demonstrating that the immunosensor is able to report objective quantitative data within clinically relevant ranges.

The multimodal measurement capabilities of the VeCare platform also enable relationships between these wound bed characteristics and bioburden parameters to be assessed. Figure 5B shows the patient-specific correlation matrices using Pearson’s correlation coefficients between the levels of measured parameters including wound sizes. Statistically significant positive correlations between IL-6 and IL-8 levels were observed in all patients. Wound fluids reporting the presence of higher load of *S. aureus* were associated with elevated levels of IL-6 and IL-8, consistent with keratinocyte responses to *S. aureus* (*55*). The degree or extent of the correlation of wound sizes with the remaining biomarkers was patient specific, possibly owing to the samples being derived from patients of different gender, age, and wound duration. A prospective randomized study in a larger patient cohort will further confirm the prognostic value of these biomarkers for predicting wound healing status. Providing measures of inflammation and microbial bioburden that are inaccessible by current single or few noninflammatory marker sensors, the VeCare immunosensing system is anticipated to serve as a beneficial addition to the existing clinical armamentarium.

## DISCUSSION

We report the development of an integrated flexible microfluidic multiplexed immunosensing system that allows for simultaneous monitoring of multibiomarker profiles using advanced sensor layout, functionalization techniques, and wireless, flexible electronics. Here, a VeCare platform was designed to perform in situ interrogation of wound healing of venous ulcers in patients. This VeCare platform incorporated a biomarker analytical dressing consisting of a perforated wound contact layer, a microfluidic wound exudate collector, an immunosensor, and a breathable barrier into a small integrated unit that was suitable for direct application onto the skin wound. A biomimetic passive microfluidic collector was developed to facilitate accurate and efficient determination of clinically relevant fluids in situ. A directional liquid transport system formed by a polar array of interconnected half-open, sawtooth-shaped capillary channels with decreasing width facilitated efficient wound fluid accumulation for wound fluid analysis. The immunosensor array delivered simultaneous quantitative assessment of multiple clinically relevant biomarkers (TNF-α, IL-6, IL-8, TGF-β1, pH, and temperature), and also bioburden (*S. aureus*) within minutes. The cytokine sensors characteristically exhibited selectivity, specificity, and reproducibility with minimal interference. The pH sensor was characterized to exhibit linearity, repeatability, and reproducibility. The immunosensor array was scalable and readily adjusted to fulfill a variety of potential applications. The immunosensor revealed the capability of in situ multibiomarker assessment and biocompatibility in wound mice models. A portable wireless analyzer was also designed to interface with the immunosensor. Last, an accompanying application containing a GUI to assist the management of patient’s profiles and medical records while facilitating data collection, analysis, and visualization was developed to integrate the VeCare immunosensing platform with existing patient records and enables rapid on-site clinical decisions to be made. As a proof of principle, the VeCare was applied to assess wound exudates collected from patients with nonhealing venous ulcers, once a week for five consecutive weeks. A graphical depiction of clinically relevant indicators of healing and bioburden served as a combined diagnostic/prognostic tool for better and more precise clinical management of the patient and their wounds. We reported the analysis of patient-specific correlation matrices, identifying relationships among measured parameters, including wound size. The VeCare platform delivers rapid point-of-care delivery of the multiple quantitative clinical measurements. We believe that the VeCare platform represents the first of its class of functioning of point-of-care devices and is able to deliver accurate and relevant personalized clinical diagnostic information to address the unmet need of the multitude of individuals suffering from nonhealing chronic ulcers. Simplicity in design allows the VeCare platform to be robust, adaptable, and customizable. Furthermore, we propose that our sensor technology allows alternative panel of biomarkers for a variety of applications requiring multiplexed analyses, for example, in diagnostic pathology and high-content screening. The VeCare platform is readily reconfigured to detect other skin bacteria (e.g., *Enterococcus faecalis*, *Pseudomonas aeruginosa*, *Staphylococcus epidermidis*, and *Corynebacterium* spp.), enabling pathogen-infected wounds to be stratified from wounds colonized by commensal organisms. Future work now includes integrating the immunosensor into a smart dressing that meets the safety, regulatory, and mass production considerations, as well as exploring the utilization of sensor data in existing clinical workflows. The limited clinical data of the study were due to the sample size and visit frequency of patients with venous ulcer over the duration of study. A larger prospective randomized clinical trial will be undertaken on patients with different types of nonhealing chronic ulcers (e.g., venous ulcers, diabetic foot ulcers, and pressure ulcers) to confirm the usefulness and clinical value of the VeCare technology platform in a primary care setting.

## MATERIALS AND METHODS

### Materials

Iron(III) chloride (FeCl_3_), *N*,*N*-dimethylformamide (DMF), tetraalkylammonium (TAA), *N*-methyl-2-pyrrolidone (NMP), potassium hexacyanoferrate(III) [K_3_Fe(CN)_6_], potassium hexacyanoferrate(II) trihydrate [K_4_Fe(CN)_6_·3H_2_O], potassium chloride (KCl), tris(2-carboxyethyl)phosphine hydrochloride (TCEP), MCH, hydrochloric acid (HCl), aniline, disodium phosphate (Na_2_HPO_4_), citric acid, buffer solution pH 10.00 Certipur, human serum, and human plasma were purchased from Sigma-Aldrich. Polyethylene terephthalate (PET) films (ST506) were purchased from MELINEX. Bulk graphite crystals (purity 99.9%) were purchased from HQ Graphene. Organic spherical gold nanoparticles (diameter of 20 nm, dispersed in DMF) were purchased from Nanopartz Inc. The modified oligos and TE buffer (10 mM tris and 0.1 mM EDTA) were purchased from Integrated DNA Technologies. Recombinant human TNF-α, IL-6, IL-8, TGF-β1, IL1β, IL-2, IL-7, interferon-γ, recombinant mouse TNF-α, and bovine serum albumin were purchased from R&D Systems. Phosphate-buffered saline (PBS) (1×) without calcium and magnesium was purchased from Lonza. *S. aureus* HG001 was provided by the Department of Microbiology and Immunology at National University of Singapore. UltraPure deoxyribonuclease (DNase)/ribonuclease (RNase)–free distilled water, BD Difco LB broth, Miller (Luria-Bertani), and oxoid tryptone soya agar (TSA) were purchased from Thermo Fisher Scientific. RAPID´*Staph* agar and egg yolk with potassium were purchased from Bio-Rad.

### Simulation and experiments of the directional liquid transport system

Computational fluid dynamics module (COMSOL Multiphysics 5.3a, Two-Phase Flow, Level Set interface) was used to simulate the directional liquid transport system. The experimental process was recorded using a high-speed camera (FASTCAM Mini AX, Photron) mounted on an inverted microscope system (IX71, Olympus) with the settings of 125 frames per second, 1024 × 1024 resolution (movie S8).

### System integration of the VeCare platform

The base electrode patterns were designed using AutoCAD 2018. To fabricate the base electrodes on a dressing, a sacrificial layer of Ni (25 nm) was deposited on a Si wafer using a sputter (ATC-2200 UHV, AJA). A bottom insulation layer of SU-8 3025 (~20 μm) was spin-coated on the Ni layer and patterned by photolithography. Cr/Au (30 nm/50 nm) was deposited and patterned on the SU-8 layer using a thermal evaporator (NANO 36, Kurt J. Lesker). Ag (200 nm) was deposited on the reference electrode area (diameter of 3 mm) using an e-beam evaporator (AJA). A top insulation layer of SU-8 3025 was spin-coated followed by photolithography to expose the working area of the electrochemical electrodes, providing a microwell for each working electrode. FeCl_3_ solution (0.1 M) was dropped on top of Ag for 1 min to generate the Ag/AgCl reference electrode. The microfluidic wound exudate collector was formed by spin-coating a layer of SU-8 2150 (~150 μm) on top of the insulation layer and patterned by photolithography. The Ag/AgCl reference electrode was temporarily protected by a layer of 950 PMMA A4 (2 μm). The entire stack was released from the Si wafer after the Ni layer was etched by 30% FeCl_3_ solution. Next, following the removal of the poly(methyl methacrylate) (PMMA) layer, the electrodes were transfer-printed to a medical-grade polyurethane film (Tegaderm). Alternatively, the base electrodes can be fabricated on a PET film (125 μm). After the AuNPs-GP modification and the aptamer immobilization, the immunosensor was lastly capsulated by a perforated medical-grade polyurethane film as the wound contact layer. A portable wireless analyzer was fabricated to interface with the electrode arrays of the immunosensor. A mobile device application containing a GUI was developed to accompany the wireless analyzer.

### Preparation and characterization of the AuNPs-GP nanocomposite

The graphene flakes were firstly prepared via cathodic exfoliation. Briefly, the electrochemical exfoliation of bulk graphite was performed using an electrochemical workstation (CHI 760E) consisting of a two-electrode system. Bulk graphite crystals were placed as the working cathode, and a Pt wire was used as the counter electrode. A nonaqueous solution consisting of 0.01 M TAA salt and NMP was used as the electrolyte. The expansion of bulk graphite was achieved using a cathodic voltage of 8 V. The expanded graphene flakes were further exfoliated and isolated using centrifugation and dried. The dried graphene flakes were dispersed in DMF (1.4 mg ml^−1^) followed by ultrasonication of 3 hours using an ultrasonic cleaner (SW1, Sonoswiss AG). Excessive AuNPs dispersion was added into the graphene dispersion followed by sonication of 1.5 hours to create well adsorption between graphene and AuNPs. The dispersion further underwent a centrifuge (Heraeus Pico 17, Thermo Fisher Scientific) at 13,000 rpm for 5 min followed by washing with DMF. This step was repeated for several times to remove the unadsorbed AuNPs. The dispersion was lastly sonicated for 5 min to obtain the AuNPs-GP nanocomposite. The composite is stored at 4°C when not in use and was sonicated for 5 min before each use. The morphology images of the AuNPs-GP were acquired using a FESEM (Verios 460, FEI). The Raman spectra were measured using a Raman microscope (Alpha 300R, Witec).

### Aptamer sequences

The TNF-α aptamer sequence is 5′-/5MeBlN/rG*rG*rA*rG*rU*rA*rU*rC*rU*rG*rA*rU*rG*rA*rC*rA*rA*rU*rU*rC*rG*rG*rA*rG*rC*rU*rC*rC/3ThioMC3-D/-3′ (*28*). Specifically, the RNA oligo was modified with a disulfide (S─S) bond at the 3′ terminus through a three-carbon (C3) spacer, and MB at the 5′ terminus through an amino modifier. Phosphorothioate bonds (marked with *) were introduced to inhibit RNA from RNase degradation.

The novel IL-6 aptamer sequence is 5′-/5ThioMC6-D/GGTGGCAGGAGGACTATTTATTTGCTTTTCT/3MeBlN/-3′ (*29*). Specifically, the DNA oligo was modified with a S─S bond at the 5′ terminus through a six-carbon (C6) spacer, and MB at the 3′ terminus through an amino modifier.

The novel IL-8 aptamer sequence is 5′-/5ThioMC6-D/rGrGrGrGrGrCrUrUrArUrCrArUrUrCrCrArUrUrUrArGrUrGrUrUrArUrGrArUrArArCrC/3MeBlN/-3′ (*30*). Specifically, the RNA oligo was modified with a S─S bond at the 5′ terminus through a C6 spacer, and MB at the 3′ terminus through an amino modifier.

The TGF-β1 aptamer sequence is 5′-/5MeBlN/CG*CTCGG*CTTC*ACG*AG*ATT*CGTGT*CGTTGTGT*C*CTGT*A*C*C*CG*C*CTTG*A*C*C*AGT*C*ACT*CT*AG*AGC*AT*C*CGG*A*CTG/iSpC3//3ThioMC3-D/-3′ (*31*). Specifically, the DNA oligo was modified with a S─S bond at the 3′ terminus through a C3 spacer, and MB at the 5′ terminus through an amino modifier. An internal spacer C3 was incorporated to lengthen the spacer arm. Phosphorothioate bonds were introduced to inhibit DNA from DNase degradation.

The novel *S. aureus* aptamer sequence is 5′-/5ThioMC6-D/TCGGCACGTTCTCAGTAGCGCTCGCTGGTCATCCCACAGCTACGTC/3MeBlN/-3′ (*32*). Specifically, the DNA oligo was modified with a S─S bond at the 5′ terminus through a C6 spacer, and MB at the 3′ terminus through an amino modifier.

The novel mouse TNF-α aptamer sequence is 5′-/5ThioMC6-D/GCGCCACTACAGGGGAGCTGCCATTCGAATAGGTGGGCCGC/3MeBlN/-3′ (*57*). Specifically, the DNA oligo was modified with a S─S bond at the 5′ terminus through a C6 spacer, and MB at the 3′ terminus through an amino modifier.

### Preparation of the pH sensor

The base electrodes were firstly cleaned with acetone and ethanol using the ultrasonic cleaner, respectively. To electropolymerize the PANI layer, 0.1 M aniline/0.1 M HCl solution was dropped on the entire electrochemical operation area, followed by CV from −0.2 to 1 V for 25 cycles at 100 mV s^−1^ using a potentiostat (CompactStat.h, Ivium, hereinafter the same).

### Preparation of the aptasensors

Following the pH sensor preparation, the electrodes were firstly rinsed with copious sterilized ultrapure (Milli-Q) water (hereinafter referred to as “ultrapure water”) and dried under N_2_. Then, AuNPs-GP dispersion was drop-casted onto each working electrode and dried. The AuNPs-GP–modified working electrodes were rinsed with copious ultrapure water, followed by a second wash with DNase/RNase-free distilled water (hereinafter referred to as “distilled water”). Oligos (100 μM) were reduced by 10 mM TCEP at room temperature for 1 hour to cleave the S─S bond. Consecutively, the oligos were then diluted to 10 μM using TE buffer and vortexed for 10 s to help disperse. Ten micromolar of TNF-α, IL-6, IL-8, TGF-β1, and *S. aureus* aptamer dispersion was dropped onto each working electrode respectively and incubated airtight at room temperature for 6 hours. The aptamer-immobilized electrodes were subsequently rinsed with copious ultrapure water, followed by a second wash with distilled water. Three millimolar of MCH was dropped onto each working electrode and incubated airtight at room temperature overnight. Last, after being rinsed with copious ultrapure water, followed by a second wash with distilled water, the aptasensors were ready for use.

### Wireless electrochemical analyzer

Drawing inspiration from the open-source universal wireless electrochemical detector (UWED) (*58*), we have designed and fabricated a wireless electrochemical analyzer to carry out comprehensive chronic wound monitoring. Unlike the UWED, which was designed to operate on a single channel with a commercial three-electrode cell, our device is a multichannel design, capable of performing multiple analysis techniques with our 10-electrode sensor.

#### Hardware design

The main components include the digital-to-analog converter (DAC), low-pass filter, potentiostat, analog switches, multiplexer, temperature-sensing circuit, and, lastly, the microcontroller RFduino. The hardware was made to interface with the immunosensor described in previous sections. Using Autodesk Eagle 9.3.0, the circuit schematics were first designed, as shown in fig. S11. Subsequently, we designed the component layouts and wires for the two-layer PCB prototype and the FPCB, shown in fig. S12. Both designs were fabricated and partially assembled using a turnkey PCB service (Interhorizon Corporation Pte. Ltd., Singapore). We inspected the PCBs with a microscope and a multimeter to ensure correct connections between all the components (table S2).

#### Microcontroller, DAC, filter, and potentiostat

We adopted the microcontroller, DAC, filter, and potentiostat from the UWED. At the core of our device, we use the microcontroller RFduino, which is a low-cost 32-bit ARM processor. The RFduino chip package incorporates many general purpose input-output (GPIO) ports and an I2C bus for interfacing with peripheral components, as well as an on-board 10-bit analog-to-digital converter for the sampling of measurement data. Furthermore, it is compatible with the Arduino developing environment and has an integrated BLE front-end to communicate with a BLE-enabled mobile device such as a phone or tablet. The DAC receives digital voltage input from the microcontroller via the I2C protocol and outputs analog voltage to the working electrode (WE) and reference electrode (RE) through two separate channels. A second-order low-pass filter using operational amplifiers (op amp) was included to minimize electrical noise in the RE potential. WE potentials are set directly from the second output of the DAC.

#### Analog switches and multiplexer

To achieve multitechnique and multichannel operations, we used analog switches and a multiplexer integrated circuit (IC) component (triple three-to-one multiplexer IC, Analog Devices Inc., ADG793G) to realize the necessary hardware logic, controllable using GPIO and I2C protocol, respectively. The analog switches are important to enable switching between different techniques. To support both amperometric (SWV) and potentiometric (OCP) measurements, we use a set of two switches (S1 and S2; Fig. 1F) to alternate the op amp OP2 between a transimpedance amplifier and a voltage follower configuration. Additional switches are used to realize the necessary hardware logic to support switching between OCP, SWV, and temperature measurement, while the multiplexer IC allows for a programmable selection of each WE channel.

#### Temperature-sensing circuit

The temperature-sensing circuit was designed on the basis of the Wheatstone bridge differential amplifier configuration, which enables accurate measurement of resistance. The Wheatstone bridge makes use of two voltage divider paths to establish a balancing point, at which a small deviation in the resistance of the temperature sensor would produce a differential voltage at the output of the op amp. The balancing resistor was chosen to be 2.26 kilohm, and the instrumentation amplifier AD627ARZ configured to a gain of 25 was used.

#### Custom MATLAB application

A mobile application was developed using MATLAB 2018b to accompany the VeCare, providing a GUI as well as comprehensive data processing and reporting. It can be run on any personal computer or mobile tablet that supports MATLAB 2018b, with the help of a BLE-to-USB adaptor. The application was designed as a one-stop patient management, data recording, data analysis, and result visualization system, intended for use by the health care provider. Upon turning on the VeCare, a BLE connection was established to the application using universally unique identifiers. Thereafter, the health care provider can use the application to manage patient profiles, collect sensor data, obtain visual feedback from the GUI in real time, analyze the data and generate useful results, as well as record them to the respective profiles for monitoring over an extended time period. Screenshots of the relevant tabs in the GUI are shown in fig. S13. A demonstration of the mobile application was recorded in movies S9 to S11.

#### Power source

The VeCare hardware can be powered by a single rechargeable 3.7-V lithium-ion polymer battery with the desired capacity. In our implementation, a battery pack of 190-mAh capacity was used (fig. S12H), which provides an estimated 40 hours of continuous active operation. Being a point-of-care diagnostic device, the actual battery life may be significantly longer, depending on how often it needs to be active. Low-dropout regulator ICs (MICREL MIC5205-3.3YM5) were used to produce separate 3.3-V digital and analog power supplies, respectively serving the RFduino microcontroller and the analog peripheral components, creating separate digital and analog circuitry to prevent digital noise from degrading analog performance.

### Qualitative analysis and characterization of the immunosensor

The qualitative assessment of aptasensors was conducted in 5 mM K_3_Fe(CN)_6_/K_4_Fe(CN)_6_ (1:1) containing 0.1 M KCl dropped on the entire electrochemical operation area. For CV measurement, the potential range is from −0.6 to 0.6 V at different scan rates (150 to 10 mV s^−1^) for the quasi-reversibility analysis of the AuNPs-GP–modified electrode, while the scan rate is fixed at 50 mV s^−1^ for the stepwise assembly analysis. For EIS measurement, the applied potential is 0.2 V. Voltage frequencies range from 100 kHz to 0.01 Hz with an amplitude of 5 mV. The Randles circuit was used to fit the Nyquist plots.

The cytokine sensors were characterized in human serum with spiked analytes (reconstituted in PBS). Bacteria culture and enumeration were conducted before the characterization of *S. aureus*. Briefly, an *S. aureus* colony from a streak plate was inoculated into 10 ml of sterilized LB broth (hereinafter referred to as “LB broth”) and allowed to grow at 37°C for 17 hours at 200 rpm. One milliliter of the inoculum and its serial diluent was respectively mixed with 15 ml of sterilized TSA medium for pour plate culture. After incubation, the plate with visible isolated colonies showing between 30 and 300 was used to estimate the *S. aureus* cell density in the original inoculum. Subsequently, *S. aureus* was pelleted after being centrifuged at 4000 rpm for 5 min and reconstituted in human serum for sensor characterization. The SWV measurement for aptasensors was scanned from −0.8 to 0 V with a step potential of 4 mV. The frequency is 50 Hz with a pulse amplitude of 40 mV. In addition, the solutions with pH values of 3.76, 4.56, 5.47, 6.2, 7.06, and 8.04 used for pH sensor characterization were prepared by a mixture of serum and McIlvaine buffer, while the solution with pH 9.01 was obtained by a mixture of serum and pH 10.00 buffer. The temperature sensor was characterized in a glass beaker containing water whose temperature was tuned by a hotplate underneath the beaker.

The long-term stability of the aptasensors or pH sensor was studied by observing the longitudinal weekly variations of the peak height against no analyte or OCP at pH 7.06 for four consecutive weeks. The immunosensor was stored airtight and at 4°C in between each measurement.

### In situ wound monitoring and biocompatibility study in mice models

#### Characterization of the mouse TNF-α sensor

Figure S14A shows the performance of the aptasensor against analyte with different concentrations. The peak current height was observed to decrease with increased target concentration. The relative reduction of peak height normalized to peak height against no analyte with analyte concentration is exhibited in fig. S14B. Notably, the concentration range of mouse TNF-α (0 to 1800 pg ml^−1^) was based on levels reported in wound tissues from mice models (*50*).

#### Animal procedures

All animal procedures were performed under protocol number A0367, approved by the Institutional Animal Care and Use Committee of the Animal Research Facility of Nanyang Technological University. Mice were housed in individual ventilated cages over a 12-hour light/dark cycle. They were fed a standard laboratory diet and water ad libitum. In this study, male Institute of Cancer Research (ICR) outbred mice (IcrTac:ICR, provided by InVivos, Singapore) 10 to 12 weeks of age, 25 to 35 g in weight were used. Inhaled isoflurane (5% mg kg^−1^) was used to induce anesthesia, which was checked by testing pedal reflex. The back of the animal was prepped by shaving with an electric hair trimmer, with care not to induce any trauma with razor teeth. Depilatory cream was then applied to the shaved skin for 2 min. The hair and cream were removed with warm water and gauze. Clean dry gauze was used to wipe off all remaining hair remover cream to insure no risk of skin irritation or lesions. Animals were injected subcutaneously with buprenorphine (1.5 mg kg^−1^) before wounding and daily for 3 days after wounding and on days of sensor placement. To produce full-thickness excisional wounds on day 0, the back skin of the mice was lifted away from the dorsum and a 6-mm biopsy punch used to incise and perform the wound through the panniculus carnosus. This technique was used to produce two bilateral wounds equidistant from the midline and spaced either side of the dorsum. A clean dry gauze was used to remove any blood resulted from the surgical procedure. Only when the bleeding stopped, which happened quite quickly in the mice, was the gauze removed. An immunosensor (diameter of 8 mm) was placed on either wound, while the other wound was used as a control. The wound with sensor contact and the control were randomly assigned. The immunosensor or the control was dressed with small individual sections of a Tegaderm film. A large single dressing of OPSITE (Smith and Nephew) was then used to cover the whole back. The immunosensor was left in place for 1 hour with the animals allowed to recover in their normal housing, before readings were taken under anesthesia. An estimated volume of 5 μl of wound exudate was accumulated in a flow rate up to 0.43 mm^3^ s^−1^, which was sufficient for sensor readings (fig. S8A). Before measurement, the stabilization of the signals was confirmed when the peak height variation of three continuous scans was under 1% (noise level). The immunosensor was removed after use and the dressing replaced.

#### Tissue processing sectioning and staining

Animals were euthanized via CO_2_ inhalation, with cervical dislocation used as a secondary means to confirm death. Animals were culled at 1, 3, and 5 days after wounding (*n* = 3 per time point). The back skin was excised in one large piece and laid flat on a smooth card. Wounds were excised and fixed in 4% paraformaldehyde for at least 24 hours and stored at 4°C. After fixation, samples were transferred to 70% (v/v) ethanol for 24 hours before processing in a HistoCore PEARL (Leica) tissue processor. Tissues are put through an ethanol concentration gradient (70, 80, 95, and 100% × 3, 45 min, 45°C) followed by xylene (3 × 45 min, 45°C) and paraffin (3 × 45 min, 62°C) and then transferred to a paraffin wax tissue embedder (HistoCore Arcadia C and H, Leica). Then, 4-μm tissue sections were produced using a Leica RM2245 microtome (Leica) and attached on Polysine slides. These were dried at 40°C for at least an hour before staining. H&E staining was achieved using a Leica Autostainer XL (Leica). Organo mounting medium (Sigma-Aldrich) was used to mount slides.

#### Bright-field microscopy and image analysis

Images of the wounds were taken daily after sensor reading or during dressing changes using a camera (Nikon D5600) with a scale and color reference. Wound area measurements were obtained using ImageJ (National Institutes of Health). H&E-stained tissues were imaged using an Axio Scan.Z1 slide scanner with 20× objective (Zeiss). Images were qualitatively examined and exported using ZEN (Zeiss). Further quantitative analysis was performed on exported images using ImageJ. Epidermal thickness was measured at 150 μm back from the leading edge on both sides of each wound and values averaged for each sample. Reepithelialization distance measurement for each sample was obtained by measuring the length of nascent epidermis outgrowing from the wound edge on both sides of each wound using ImageJ and averaged. Infiltrating immune cells were qualitatively assessed by looking at polymorphonuclear cells and macrophage cells in a region of interest in the dermis at the wound edge on both sides of each wound.

### Clinical study on the wound exudates from patients with venous ulcer

The study was conducted in Singapore General Hospital (SGH) and approved by the SingHealth Centralized Institutional Review Board A (Ref: 2018/3061). Participants are patients who were diagnosed with venous ulcers and treated with four-layer compression bandaging. Their wounds were managed by the vascular surgery team at SGH. Patients aged above 21 with an ankle-brachial pressure index ≥ 0.8 and open ulceration between the ankle and knee that failed to reduce in the size for more than 12 weeks were considered eligible to participate. Five patients (three male and two female; age range, 57 to 77) were recruited from the vascular outpatient clinics and have given their consent to participate. The wound exudates were collected once a week at their scheduled weekly change of dressing for five consecutive weeks using a standard protocol (*6*, *19*). Briefly, upon the removal of the four-layer bandages, the wound was cleansed with normal saline and covered by a transparent film dressing. The corresponding leg was kept dependent in the seated position for ~40 min. The accumulated wound exudates from all wounds of the affected leg were aspirated out from the transparent film dressing using a hypodermic needle and syringe. In view of vascular leakage in post-conservative debridement that might compromise biomarker profiling, wound fluid sampling was conducted before conservative debridement. The ulcer size was measured using a ruler method. Topical dressings impregnated with antiseptic (e.g., cadexomer iodine and nanocrystalline silver) were used for wounds with colonization. They were placed onto the wounds after conservative debridement and before fresh four-layer bandages were applied. Foam dressings were placed underneath the bandages for wounds with excessive exudates where applicable.

Rapid and simultaneous assessment of the wound bed characteristics and bioburden biomarkers in the wound exudates was conducted using the VeCare. On the other hand, the wound exudates were also analyzed using conventional methods. Briefly, to conduct the cytokines assessment, a multiplex ELISA kit was custom-made by Thermo Fisher Scientific and was performed according to the manufacturer’s instruction on neat samples. The reading was done using Luminex 200 with xPONENT 3.1, and the concentration of cytokines was determined using MasterPlex QT 2.0.0.59. In addition, the pH values of the wound fluid samples were measured using a commercial pH meter (LAQUAtwin pH-33, HORIBA). *S. aureus* was detected via coagulase-positive *Staphylococci* enumeration. Briefly, 10 μl of wound exudate was first diluted in 10 ml of LB broth, followed by serial dilution. One milliliter of the original diluent and its serial diluent was respectively mixed with 15 ml of sterilized RAPID´*Staph* agar medium, a mixture of base medium and egg yolk with potassium, for pour plate culture. After incubation, *S. aureus* formed black colonies on the opaque medium with a clear halo around the colonies attributed to egg yolk proteolysis. The number of *S. aureus* colonies on a RAPID´*Staph* agar plate showing between 30 and 300 was used to estimate the *S. aureus* cell density in the wound exudate sample. Optical images of the RAPID´*Staph* agar plates for wound fluid assessment of patients 1 to 5 are as shown in figs. S15 to S19.

### Statistical analyses

GraphPad Prism 8 and R (version 3.6.1) were used to perform statistical tests and data visualization. When comparing two groups, Wilcoxon signed-rank test was used. *P* < 0.05 was considered as significant. The R packages ggplot2 (version 3.0.0) (*59*) and GGally (version 1.4.0) (*60*) were used to plot the correlation matrices.

## SUPPLEMENTARY MATERIALS

Supplementary material for this article is available at http://advances.sciencemag.org/cgi/content/full/7/21/eabg9614/DC1
